# International Expert Consensus on Relevant Health and Functioning Concepts to Assess in Users of Tobacco and Nicotine Products: Delphi Study

**DOI:** 10.2196/58614

**Published:** 2025-01-02

**Authors:** Vivienne Law, Esther F Afolalu, Linda Abetz-Webb, Lee Andrew Wemyss, Andrew Turner, Christelle Chrea

**Affiliations:** 1 Zebra26 Ltd. Devon United Kingdom; 2 PMI R&D, Philip Morris Product S.A. Neuchâtel Switzerland; 3 Patient-Centered Outcomes Assessments Ltd. Cheshire United Kingdom; 4 DaSH Global Cheshire United Kingdom

**Keywords:** Delphi study, expert consensus, outcome measures, health and functioning, tobacco and/or nicotine products

## Abstract

**Background:**

A Delphi study was conducted to reach a consensus among international clinical and health care experts on the most important health and functioning self-reported concepts when evaluating a switch from smoking cigarettes to using smoke-free tobacco and/or nicotine products (sf-TNPs).

**Objective:**

The aim of this research was to identify concepts considered important to measure when assessing the health and functioning status of users of tobacco and/or nicotine products.

**Methods:**

Experts (n=105), including health care professionals, researchers, and policy makers, from 26 countries with professional experience and knowledge of sf-TNPs completed a 3-round, adapted Delphi panel. Online surveys combining quantitative (MaxDiff best-worst scaling and latent class analysis) and qualitative assessments were used to rank and achieve alignment on the importance of 69 health and functioning concepts. All experts participating in round I completed round II, and 101 (95%) completed round III.

**Results:**

The round I analysis identified 36 (52%) out of 69 concepts that were refined for the round II assessment. The highest-ranked concepts reflected health-related impacts, while the lowest-ranked ranked concepts were related to aesthetics and social impacts. Round II ranking reinforced the importance of concepts relating to health impacts, and the analysis resulted in 20 concepts retained for round III assessment. In round III, the 4 highest-ranked concepts were cardiovascular symptoms, shortness of breath, chest pain, and worry about smoking-related diseases and impact on general health, and they made up 50% of the total score in the MaxDiff analysis. Experts reported likelihood of seeing measurable levels of change in the final 20 concepts with a switch to an sf-TNP. The majority of experts felt it was “likely” or “extremely likely” to observe changes in concepts such as gum problems (74/101, 73%), phlegm or mucus while coughing or not coughing (72/101, 71%), general perception of well-being (72/101, 71%), and throat irritation or sore throat (72/101, 71%). Latent class analysis revealed subgroups of experts with different perceptions of the relative importance of the concepts, which varied depending on professional specialty and geographic region. For example, 74% (14/19) of oncologists aligned with the subgroup prioritizing physical health symptoms, while 71% (12/17) of experts from Asia aligned with the subgroup considering both physical health and psychosocial aspects.

**Conclusions:**

This study identified key concepts to be considered in the development of a new measurement instrument to assess the self-reported health and functioning status of individuals using sf-TNPs. The findings contribute to the scientific evidence base for understanding and evaluating both the individual and public health impacts of sf-TNPs.

## Introduction

Reducing exposure to harmful and potentially harmful constituents in cigarette smoke by cessation of smoking or switching to reduced risk tobacco and/or nicotine products (TNPs) is a major public health focus worldwide [1]. Although nicotine-replacement therapy (NRT) and targeted counseling services can help individuals achieve their quitting goals, there can be hurdles to becoming and/or staying smoking free [2].

Smoke-free tobacco and/or nicotine products (sf-TNPs) refer to TNPs that do not undergo combustion and therefore do not produce the harmful smoke generated by cigarettes, which has been established as the main risk factor of tobacco-related diseases [3]. These sf-TNPs can include heated tobacco products, e-cigarettes (vapes), other e-vapor products (e-pipes, e-cigars, and so on), nicotine pouches, and smokeless tobacco products (snuff, snus, and chewing tobacco) [3]. Regulatory bodies, like the US Food and Drug Administration (FDA), require robust data to evaluate the health impacts of sf-TNPs and guide regulatory decisions on product authorization. For instance, the FDA’s modified risk tobacco product pathway mandates comprehensive scientific evidence to support claims that a tobacco product reduces harm or the risk of tobacco-related disease in individual tobacco users and benefits the health of the population as a whole [4]. Some sf-TNPs have been recognized as modified-risk tobacco products under this pathway [4,5] and may help reduce the negative health effects associated with commercially marketed combustible tobacco products such as cigarettes [6-8].

The adverse effects of smoking on health have been well documented, whereas stopping smoking can improve health and reduce the risk of disease [6,9,10]. Evidence also suggests that switching away from cigarettes to using sf-TNPs can help reduce cigarette consumption and may lead to cessation in some cases [8]. While studies have shown that sf-TNPs reduce exposure to many of the harmful and potentially harmful constituents found in cigarette smoke, they may still pose health risks. For instance, e-cigarettes, heated tobacco products, and smokeless tobacco have been associated with some cardiovascular, respiratory, and oral health risks, albeit to a lesser extent than combustible cigarettes [11-14]. The long-term health effects of sf-TNPs are not yet fully understood, and further research is necessary to comprehensively assess their safety profile. In addition, little is known about the self-reported health impact of switching from cigarettes to sf-TNPs [6]. In this context, measuring the self-reported experience of health and functioning (including health status, functional status, and other health-related quality of life constructs) is crucial to understanding the impact of tobacco harm reduction strategies [15].

Generic health status measures, such as the Short Form Survey–12 [16] and Short Form Survey–36 instruments [17], have shown that those who smoke tend to report lower health status compared with those who never smoked, although the impact of cessation on health status seems to be more complex. Some smoking-specific measures have been developed but not yet widely standardized; therefore, they may not be directly applicable to sf-TNPs [9,18,19]. Crucially, current measures often lack the necessary sensitivity to detect longitudinal health changes in healthy populations due to high ceiling effects at baseline [20], hindering the assessment of changes in health and functioning following a switch from smoking cigarettes to using sf-TNPs.

To help address these challenges, the development of a new self-reported measure (ABOUT – Health and Functioning) was undertaken to assess the health and functioning status of individuals using sf-TNPs. This new measure is part of the portfolio of the ABOUT Toolbox [21], consisting of self-report measures to assess perceptions and behavioral outcomes related to the use of sf-TNPs. The initial preparatory phase of the instrument development was based on several research activities (systematic literature review, reanalysis of qualitative data, and expert insights [22]) and resulted in the identification of 69 health and functioning concepts relevant to TNP use. The qualitative research phase that followed consisted of (1) concept elicitation interviews of users to understand their perceptions of health and functioning after switching to sf-TNPs [23,24] and (2) the Delphi panel study reported in this paper. The aim of the Delphi study was to identify the health and functioning concepts considered most important by clinical and health care experts (ie, health care professionals, researchers, policy makers, and those involved in smoking cessation or tobacco harm reduction) when assessing the health and functioning status of individuals who stop smoking cigarettes or switch to using sf-TNPs or NRTs. Delphi panel methodology is a well-established process for determining consensus among relevant groups of individuals to aid issue prioritization, ranking, development, and obtaining agreement on guidelines, concept-framework development, and development of outcome measures [25-27], including issues relevant to smoking-related behaviors and sf-TNP use [25,28,29]. Delphi panels have previously been used to forecast trends and changes over time in health-related matters [30-32].

## Methods

### Objectives and Summary of Approach

This Delphi panel was organized for experts to select and rank health and functioning concepts considered important to assess when individuals stop smoking combustible TNPs (eg, cigarettes) or switch to using sf-TNPs (eg, e-cigarettes [vapes], heated tobacco, or smokeless tobacco products) or NRTs. The study was conducted and reported based on available guidance for conducting and reporting Delphi studies in health care research [33].

**Figure 1:**
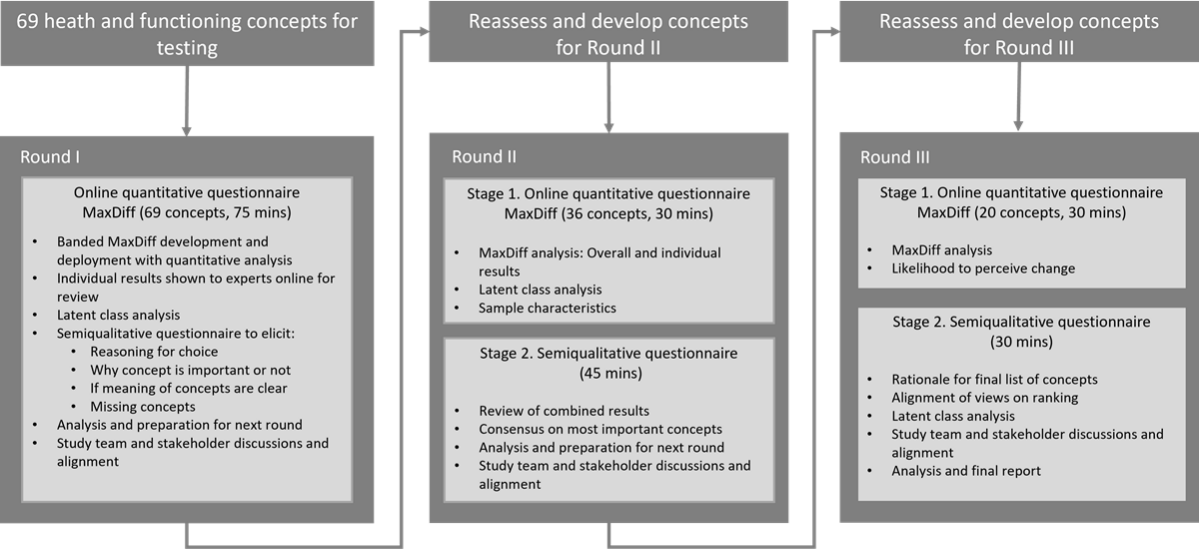
Overview of the design and structure of the adapted Delphi panel study.

A preliminary set of 69 concepts was identified from earlier research activities including a scoping literature review, results from the reanalysis of previous TNP consumer qualitative research results, and expert opinion from a small group of key opinion leaders [22]. This list was presented for evaluation in this Delphi panel according to the process described in Figure 1. An adapted approach was used for the Delphi panel, whereby the results of round I were reviewed and refined in light of the ongoing qualitative concept elicitation interview findings from consumers of TNPs and ongoing review by key opinion leaders (Supplementary Information and Table S1 in Multimedia Appendix 1). The concept list to be evaluated in round II was adjusted accordingly and refined considering the round I findings. Round III was a detailed review of the final set of health and functioning concepts. This step also included an evaluation of likelihood of change that experts considered important for each concept for inclusion in a self-reported measure of clinical relevance.

### Participants and Recruitment

For this adapted Delphi panel, we recruited experts who routinely treat, communicate with, or advise individuals who smoke or wish to stop smoking or switch to sf-TNPs. The recruitment aim was to ensure that a minimum of 100 experts globally completed all 3 rounds of the Delphi panel. To anticipate attrition between rounds, the initial recruitment target was set at 120 experts.

**Figure 2:**
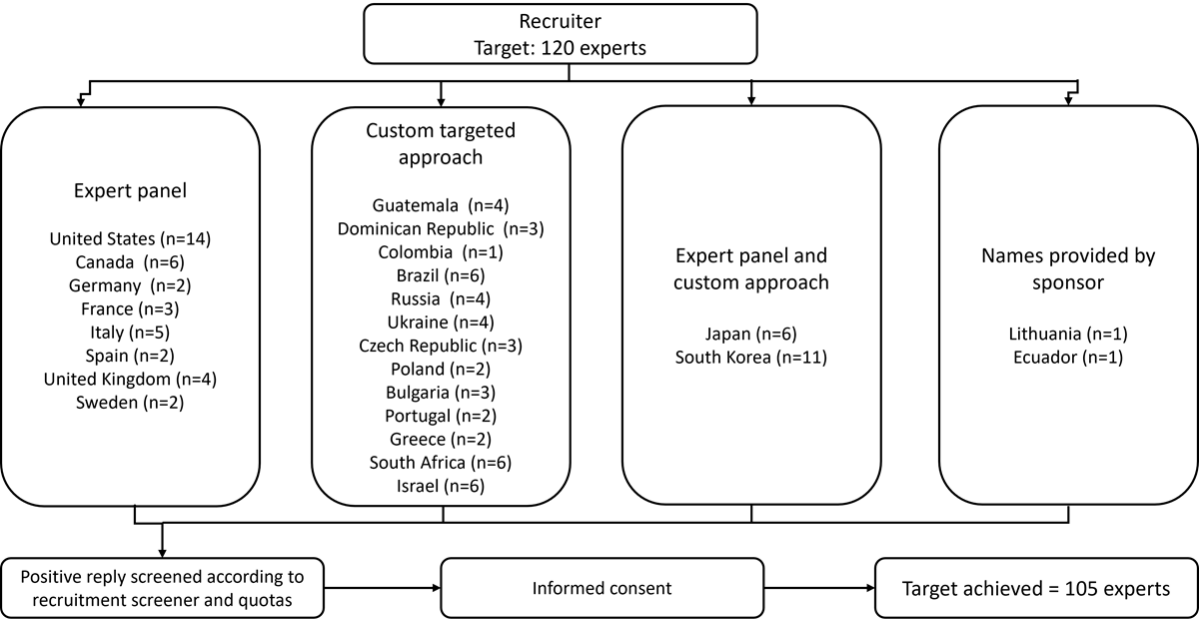
Identification and recruitment of experts through specific channels for the adapted, 3-round Delphi panel.

Recruitment was undertaken by QualWorld, who identified experts through their survey panels, existing contacts, publications, word of mouth, and canvassing through appropriate organizations in individual countries. The process and route of contact to identify potential respondents are outlined in Figure 2.

To anticipate and account for potential sensitivities among health care practitioners about undertaking a project sponsored by a tobacco company, the recruitment company sent out an initial survey asking which industries experts would be interested in working on health-related topics. Tobacco companies were listed among other industries such as mining, pharmaceuticals, and oil and gas. Only experts stating unprompted that they would consider participating in studies by tobacco companies were chosen to receive further correspondence linked to the Delphi panel. Subsequent recruitment steps proceeded on an individual basis according to predefined inclusion and exclusion criteria (Textbox 1).

To ensure consistency in responses across different regions, the Delphi panel was conducted in English where possible and in local languages for experts in Japan, South Korea, Russia, Ukraine, Czech Republic, and Bulgaria. A professional translation service translated the survey materials and responses, and back-translation techniques were used to verify accuracy. The local bilingual recruitment team members also performed proofreading and quality checks from a language and contextual perspective to ensure the translated content accurately reflected the original meaning. For each step of the Delphi panel, the English and local language surveys were programmed and uploaded for a user acceptability test by the project team before launch.

**Inclusion and exclusion criteria for the participants recruited for the Delphi panel.**

**Inclusion criteria**

- Had current clinical, academic, or professional experience in the area of smoking-related diseases, smoking cessation, nicotine addiction, or other smoking-related conditions, as well as health policy or advocacy work related to tobacco control or tobacco harm reduction
- Currently working in research, prescribing, or a recommendation capacity
- Knowledge of smoke-free tobacco and/or nicotine products or nicotine replacement therapies, works with patients to some degree, and/or recommends smoke-free tobacco and/or nicotine products or nicotine replacement therapies
- Expertise in one of the following groups:
  - Specialist physicians: smoking-related oncology; smoking-related respiratory disease, and smoking-related cardiovascular disease (specifically treatment of patients)
  - General practitioners and internal medicine physicians: those who see patients with a smoking-related condition or disease and recommend smoking cessation advice
  - Dentists or oral hygienists: those who see patients with smoking-related dental issues
  - Smoking cessation, addiction, or dependence: those who help users quit smoking with less harmful products or nicotine replacement therapy or worked with nicotine addiction; nurses undertaking smoking cessation activity; researchers in nicotine addictions; psychologists, social workers, or counselors
  - Health policy, advocacy, and non-profit organizations: smoking-related health policy, including tobacco harm reduction; organizations or charities that encourage people to quit; health policy and advocacy related to tobacco control and tobacco harm reduction. This can include physicians who work alongside the government and local policy as well as nonprofit organizations
- Ability and willingness to participate across the whole study
- Ability to complete the research in the English language (except for these countries: South Korea, Japan, Ukraine, Czech Republic, Russia, and Bulgaria)
- At least 30% of experts to identify as female (although this was not a strict quota for recruitment)

**Exclusion criteria**

- More than 50% of experts should not have been a consultant with or worked for the tobacco industry
- Had less than 5 years of clinical, academic, or professional experience
- Did not fall into one of the categories for inclusion
- Not willing to complete all 3 rounds of research

### Ethical Considerations

The New England Independent Review Board reviewed the study protocol and all study materials and any amendments and granted approval of the study (reference 1‐9184‐1).

Before participation, all participants were required to review and sign an informed consent form, which provided a detailed description of the study, outlined the procedures, and specified participant expectations. Participants had the opportunity to ask questions and receive answers before signing the consent form.

All study data were deidentified and participants were identified only by unique ID numbers throughout the research rounds.

All experts were compensated and received a cash honorarium according to local professional rates in their respective countries, ranging from £115 to £450 (approximately US $147 to US $576 at the time of the research), for their time after each round.

### Procedure and Analytical Methods

#### MaxDiff Analysis

This study incorporated the use of MaxDiff—a type of best-worst (maximum difference) scaling analytical method [34-36]. This methodology was primarily chosen due to the large number of initial concepts to be evaluated. Ranking and rating exercises with numerous concepts can lead to a cognitive burden, so a best-worst scaling approach overcomes fatigue while generating additional information by making respondents choose the most and least important concepts. The result is a list of concepts in order of relative importance [36-38].

For each round, the MaxDiff Sawtooth software algorithm [39] generated a list of scaled scores from most to least important, as well as the relative importance of one concept compared with another. This was based on the number of times that concept was rated as the most or least important. In addition, cumulative scores were used to differentiate the number of concepts accounting for 20%, 50%, and 70% of the most important concepts; the 50th percentile was used as an initial guide for which concepts to retain.

**Figure 3:**
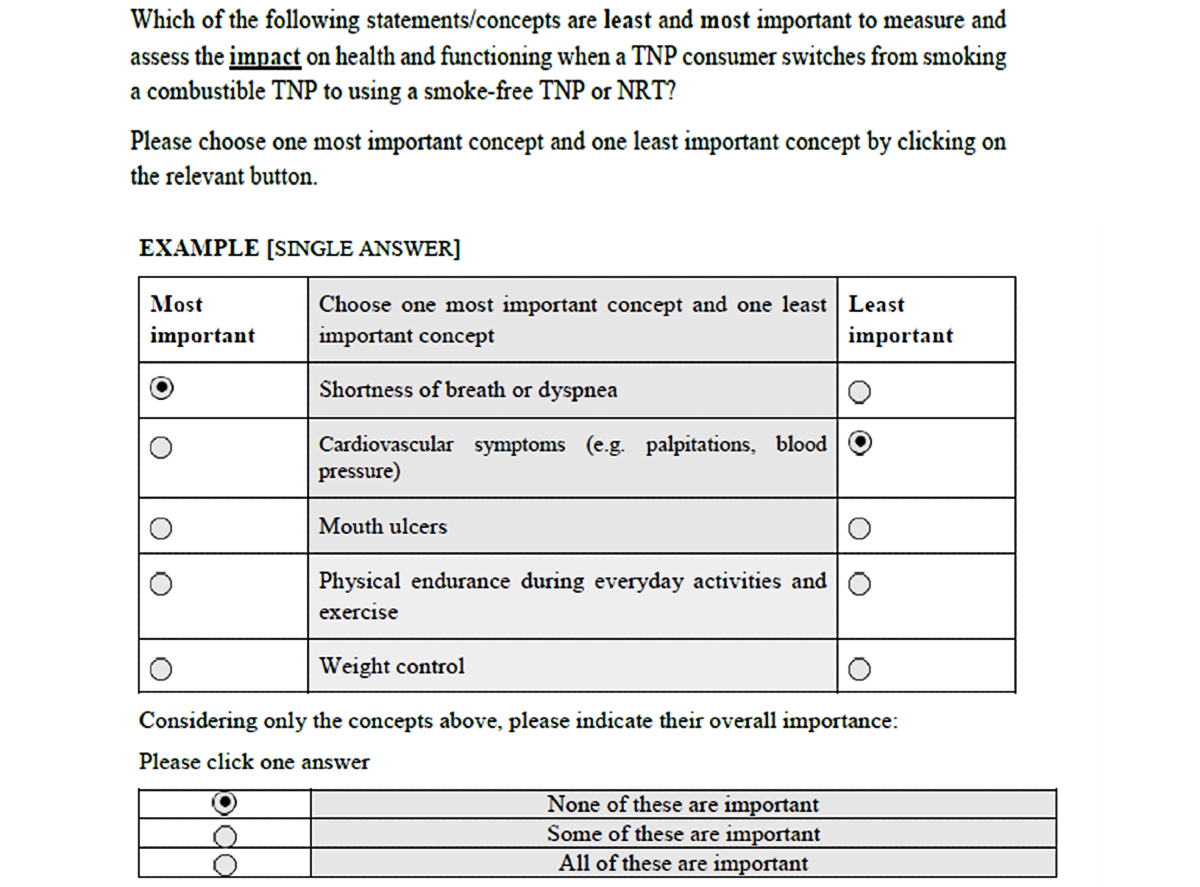
Typical presentation of the Delphi panel MaxDiff ranking question, including the anchor evaluation. NRT: nicotine replacement therapy; TNP: tobacco and/or nicotine products.

Rounds I and II also featured an “anchor” value, which was a score marking the boundary between concepts considered important (to be retained in the next round) or unimportant (to be excluded from the next round). This anchor value was derived from an anchor question representing a threshold of “important or not important” included for each concept to be assessed as above (ie, important) or below (ie, unimportant) the anchor. The anchor question in this survey required one of the following 3 responses: “none of these [items] are important,” “some of these [items] are important,” or “all of these [items] are important.” The anchor value was statistically derived using the Sawtooth software [38]. An illustration of a typical MaxDiff question is shown in Figure 3.

#### Latent Class Analysis

Latent class analysis [40] was also performed to determine whether some concepts were more important to some groups of experts over others, according to experts’ demographic or other characteristics. This ensured that the final set of items was not biased toward one subset of health care professionals at the expense of others’ preferences. Where there were concepts that were only important to subsets of the study sample but unimportant overall, these concepts could be retained.

#### Semiqualitative Assessment

Semiqualitative questions were incorporated into each Delphi panel round to provide an increased understanding of participants’ responses to the results of the ranking and scoring exercise. These questions were asked to ensure no concepts were missing or needed rewording, and to understand the reasons behind experts’ selections of importance or unimportance. They also sought to gauge agreement or disagreement with the overall ranking results and to gain insights into the reasons for these viewpoints. After each round, the semiqualitative comments were reviewed alongside the MaxDiff scores. This process involved coding and analyzing the qualitative feedback to identify common themes and insights that could provide context to the quantitative rankings. This feedback was then integrated with the quantitative MaxDiff results to refine and inform decisions about the list of concepts to be included for the next rounds. It helped explain the rationale behind the experts’ choices and highlighted any discrepancies or areas of consensus, ensuring that both qualitative and quantitative perspectives were considered in the decision-making process.

## Results

### Descriptive Participant Demographics

Approximately 3100 experts were contacted, with a final sample of 105 experts recruited across 26 countries. Table 1 shows the demographics and participant characteristics, and Table 2 shows the participant quotas per region and per specialty. Of the initial 105 experts, all participated in exploratory round I and round II, and 101 (96%) completed round III (Table 2). Nearly all participants (90/105, 87%) reported frequently addressing smoking cessation with clients or patients. A total of 64% (67/105) of experts in the study identified as male, and most were located in the Americas, Europe, or Asia. Regarding place of work, three-quarters (79/105, 75%) of experts were based in hospitals or other clinical settings, mostly as medical specialists or general care practitioners.

**Table 1:** Demographics of Delphi panel participants (rounds I-III).

Demographic variable			Respondents		
			Rounds I and II (n=105), n (%)		Round III (n=101), n (%)
**Sex**					
	Male	67 (64)		64 (63)	
	Female	38 (36)		37 (37)	
**Geographical region**					
	North America (United States and Canada)	20 (19)		17 (17)	
	Asia (Japan and South Korea)	17 (16)		17 (17)	
	Central, Caribbean, South America (Brazil, Colombia, Dominican Republic, and Guatemala)	15 (14)		15 (15)	
	Southern Europe (Spain, Portugal, Italy, Greece, and Bulgaria)	14 (13)		13 (13)	
	Western Europe (France, Germany, Sweden, United Kingdom, and the Netherlands)	13 (12)		13 (13)	
	Eastern Europe (Russia, Ukraine, and Lithuania)	9 (9)		9 (9)	
	Africa (South Africa)	6 (6)		5 (5)	
	Middle East (Israel)	6 (6)		6 (6)	
	Central Europe (Poland and Czech Republic)	5 (5)		5 (5)	
**Work setting**					
	Hospital	43 (41)		41 (41)	
	Clinical (other)	36 (34)		36 (36)	
	Professional	17 (16)		15 (15)	
	University hospital	5 (5)		5 (5)	
	University hospital; hospital setting	4 (4)		4 (4)	
**Main current role**					
	General practitioner or internal medicine	27 (26)		27 (27)	
	Oncology	19 (18)		19 (19)	
	Dentist or oral hygienist	16 (15)		15 (15)	
	Research	11 (10)		9 (9)	
	Cardiology	12 (11)		12 (12)	
	Respiratory	8 (8)		8 (8)	
	Counselor or psychologist	7 (7)		6 (6)	
	Advocacy or health policy or NGO^a^	5 (5)		5 (5)	
**Frequency of discussing smoking cessation with clients**					
	Frequent (at least weekly)	90 (86)		86 (85)	
	Not frequent (less than once a week)	11 (10)		11 (11)	
	Not applicable	4 (4)		4 (4)	
**Discussed the benefits of stopping smoking or provided information about sf-TNPs^b^or NRTs^c^to those who smoke and do not want or have difficulty quitting smoking**					
	Yes	103 (98)		99 (98)	
	No	0		0	
	Not available or prefer not to say	2 (2)		2 (2)^d,e^	
**Provided information on heat-not-burn or heated tobacco products (eg,*IQOS*, Glo) or tobacco vapor products (eg, Ploom Tech)**					
	Yes	50 (48)		46 (46)	
	No	53 (50)		53 (53)	
	Not available or prefer not to say	2 (2)		2 (2)	
**Provided information on smokeless tobacco (eg, Copenhagen Snuff, Swedish Match General Snus, Camel Snus, or any other local brands)**					
	Yes	39 (37)		35 (35)	
	No	64 (61)		64 (63)	
	Not available or prefer not to say	2 (2)		2 (2)	
**Provided information on e-cigarettes (eg, JUUL, Blu, Logic, or any other local brands)**					
	Yes	65 (62)		62 (61)	
	No	38 (36)		37 (37)	
	Not available or prefer not to say	2 (2)		2 (2)	
**Provided information on Nicotine replacement therapies (eg, nicotine gums, inhalers, nasal sprays, lozenges, patches)**					
	Yes	91 (87)		88 (87)	
	No	11 (10)		10 (10)	
	Not available or prefer not to say	3 (3)		3 (3)	
**Current or previous personal use of combustible TNPs^f^**					
	Never	65 (62)		62 (61)	
	Yes	35 (33)		34 (34)	
	Not available or prefer not to say	5 (5)		5 (5)	
**Current or previous personal use of sf-TNP or NRT of those that were currently or had used combustible TNPs**					
	Yes	12 (11)		12 (12)	

^a^NGO: nongovernmental organization.

^b^sf-TNP: smoke-free tobacco or nicotine product.

^c^NRT: nicotine replacement therapy.

^d^Not available.

^e^Data missing for one respondent in round III, stage 2.

^f^TNP: tobacco or nicotine product.

**Table 2:** Participant quotas per region and per specialty for the start of round I of the Delphi panel (N=105).

Specialty	Asia	Africa	Middle East	North America	Central America	Southern Europe	Eastern Europe	Western Europe	Central Europe
Specialist physician	6	2	3	6	7	3	4	2	6
GP^a^ or IM^b^	4	2	3	4	3	3	2	2	4
Dental specialist	3	1	0	2	2	2	3	1	2
SC^c^ or DEP^d^	2	1	0	5	2	1	3	0	3
Health policy or NGO^e^	2	0	0	3	1	0	1	0	0
Total	17	6	6	20	14	9	13	5	15

^a^GP: general practitioner.

^b^IM: internal medicine specialist.

^c^SC: smoking cessation specialist.

^d^DEP: dependence or addiction specialist.

^e^NGO: nongovernmental organization.

### MaxDiff Analysis and Semiqualitative Assessment for Reduction or Refinement of Health and Functioning Concepts

#### Round I

**Figure 4:**
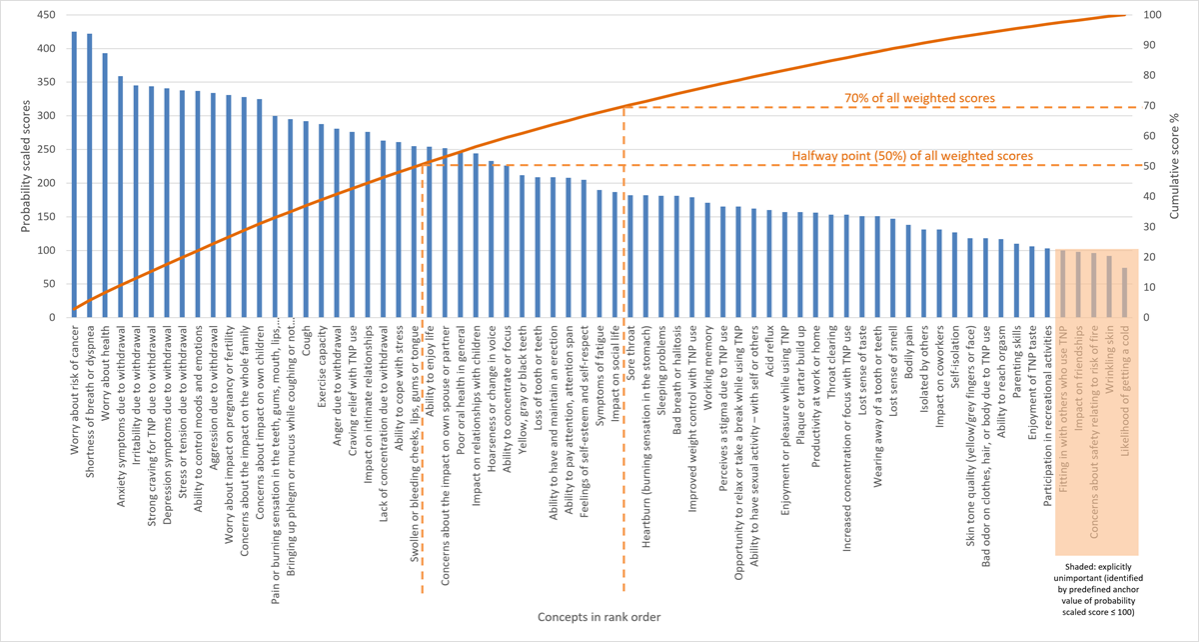
Scaling and ranking results for items considered in round I of the Delphi panel. TNP: tobacco and/or nicotine products.

A cumulative score was calculated by adding all the scaled scores (in this case, the total score was 14,900.17) and dividing by the scaled score (concept 1: 425/14,900=3%) and adding to the scaled score of the next highest concept (concept 2: 422/14,900=3%+3% concept 1 and so on; Figure 4). Regarding the relative importance of concepts in round I, when switching from combustible to sf-TNPs, experts placed importance on withdrawal symptoms, general health impacts, and emotion-related impacts. Conversely, concepts that ranked low in priority were those that referred to aesthetic concerns that would have less of a health impact or were not considered exclusive to those who smoke. Experts considered withdrawal symptoms such as anxiety and irritability as potential barriers to switching to sf-TNPs and highlighted there were levels of satisfaction (eg, enjoyment and craving relief) that those who smoke cigarettes would not want to lose when switching to sf-TNPs

When determining which concepts to include in round II of the Delphi panel evaluation, the relative score (ie, importance) for each concept was considered, resulting in 36 out of 69 concepts (52% of all concepts in ranked order) accounting for 69% (10,285/14,900) of the total importance score. Based on the results and alignment with other activities ongoing within development of the health and functioning measurement instrument, the health and functioning concepts were further reviewed for relevance and clarity, and a different, refined, reduced, and synthesized list of 36 concepts was taken forward for evaluation in round II (refer to Supplementary Information and Table S1 in Multimedia Appendix 1 for further details).

#### Round II

**Figure 5:**
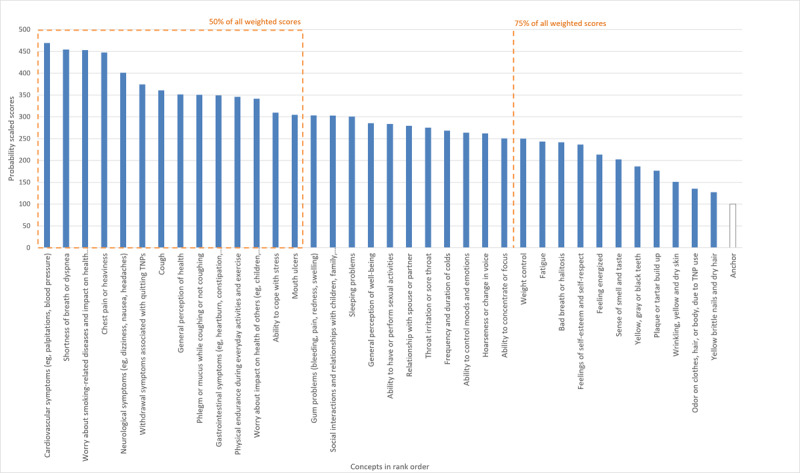
Scaling and ranking results for items considered in round II of the Delphi panel. TNP: tobacco and/or nicotine products.

Of the 36 concepts in this round, 14 made up 50% of the total scaled score (Figure 5). In general, physical health symptoms, and worries about the impact on health concepts remained the highest-ranked concepts and were considered by experts as more important than aesthetic considerations. Experts commented that concepts rated as unimportant and ranked lower in the logit analysis tended to lack specificity to smoking (ie, could be due to other causes), did not directly affect health, or were not reported frequently by TNP users. Of the 36 concepts evaluated in round II, a final selection of 20 highest-ranked concepts were identified for validation and discussion in round III.

#### Round III

**Figure 6:**
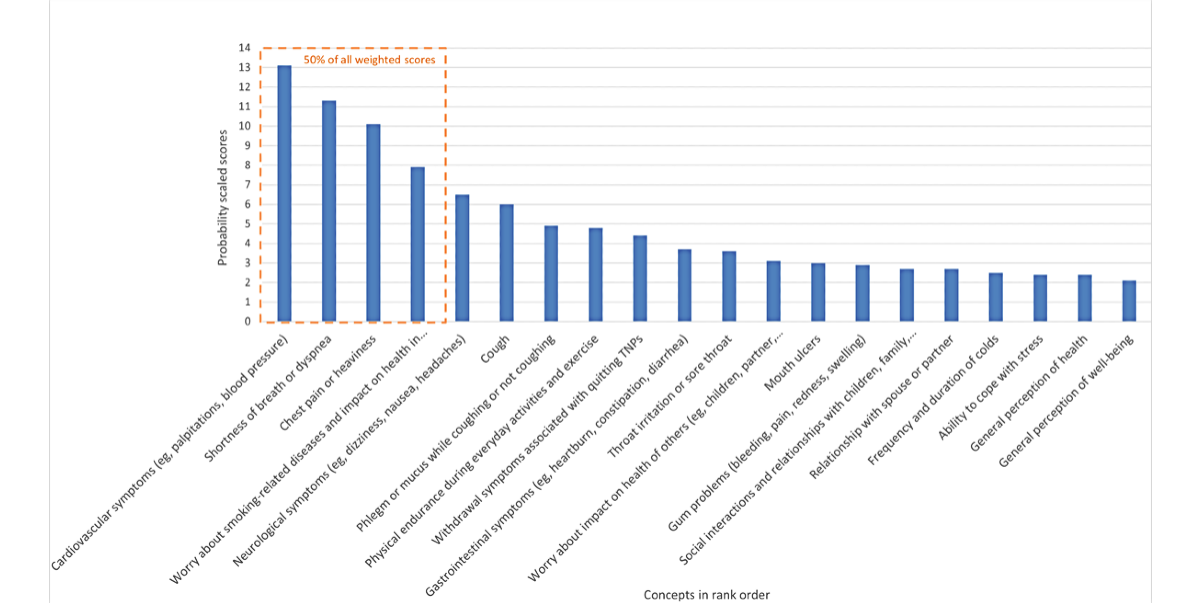
Ranking and ordering of the final 20 items identified in round III of the Delphi panel.

Of the final 20 concepts ranked in round III (Figure 6), the sum of the top 4 concept scores accounted for more 50% of the total score: cardiovascular symptoms, shortness of breath or dyspnea, chest pain heaviness, and worry about smoking-related diseases and impact on health in general. Physical health concepts remained the most important experts across all 3 rounds

### Latent Class Analysis

Latent class analysis of the results in round III revealed 2 identifiable subgroups, each with its own ranking and ordering of the final 20 concepts (Textbox 2). In both cases, concepts related to physical health, particularly cardiovascular and respiratory symptoms, ranked highest. In subgroup 1, the lowest-ranked concepts were related to psychosocial or general well-being and social interactions, and all scored equally low. For subgroup 2, there was minimal differentiation between concepts beyond the highest-ranking group.

**List of concepts in ranked order (highest to lowest) for the 2 subgroups identified by latent class analysis in round III of the Delphi panel.**

**Subgroup 1 in rank order**

- Cardiovascular symptoms (eg, palpitations and blood pressure)
- Shortness of breath or dyspnea
- Chest pain or heaviness
- Neurological symptoms (eg, dizziness, nausea, and headaches)
- Cough
- Mouth ulcers
- Phlegm or mucus while coughing or not coughing
- Throat irritation or sore throat
- Gum problems (eg, bleeding, pain, redness, or swelling)
- Gastrointestinal symptoms (eg, heartburn, constipation, and diarrhea)
- Worry about smoking-related diseases and impact on health in general (eg, cancer and stroke)
- Withdrawal symptoms associated with quitting tobacco and/or nicotine products
- Frequency and duration of colds
- Physical endurance during everyday activities and exercise
- Ability to cope with stress
- Worry about impact on health of others (eg, children, partner, family, and friends)
- Relationship with spouse or partner
- General perception of health
- General perception of well-being
- Social interactions and relationships with children, family, friends, and colleagues

**Subgroup 2 in rank order**

- Cardiovascular symptoms (eg, palpitations and blood pressure)
- Shortness of breath or dyspnea
- Worry about smoking-related diseases and impact on health in general (eg, cancer and stroke)
- Chest pain or heaviness
- Physical endurance during everyday activities and exercise
- Neurological symptoms (eg, dizziness, nausea, and headaches)
- Cough
- Worry about impact on health of others (eg, children, partner, family, and friends)
- Social interactions and relationships with children, family, friends, and colleagues
- Withdrawal symptoms associated with quitting tobacco and/or nicotine products
- Relationship with spouse or partner
- Phlegm or mucus while coughing or not coughing
- General perception of health
- General perception of well-being
- Ability to cope with stress
- Gastrointestinal symptoms (eg, heartburn, constipation, and diarrhea)
- Throat irritation or sore throat
- Frequency and duration of colds
- Gum problems (eg, bleeding, pain, redness, and swelling)
- Mouth ulcers

In stage 2 of round III, following the MaxDiff analysis, the ranked concept lists for each of the 2 latent class subgroups were shown to the experts, who were asked to identify which group best reflected their own thinking and provide a rationale for their choice. Just over half of the experts (58/100, 58%) reported that subgroup 1 best reflected their thinking compared with (42/100, 42%) for subgroup 2. When asked about their rationale for aligning with subgroup 1, the main reason was a focus on the most important physical health symptoms (33/58, 57%). The main rationale for choosing subgroup 2 was a greater emphasis on the importance of the consumer and their perspective (10/42, 24%), including psychological elements, the well-being of consumers, and impact of smoking on the health of others (14/42, 33%).

Regarding the influence of professional specialty in self-alignment with subgroups (Table 3), preference for subgroup 1 tended to be among oncologists (14/19, 74%), dentists (11/15, 73%), counselors (4/6, 67%), and general practitioners or internal medicine specialists (17/27, 63%). Conversely, all policy advisors and charity workers (5/5) felt that subgroup 2 best reflected their views. When geographical location was considered (Table 3), there was a strong preference for subgroup 2 in participants from Asia (12/17, 71%) and a slight preference among those in North America (9/17, 53%). Experts from all other regions tended mostly to identify with subgroup 1 (Middle East: 5/6, 83%; Africa: 4/5, 80%; Central Europe: 4/5, 80%; Eastern Europe: 7/9, 78%; Western Europe: 9/13, 69%; Southern Europe: 8/13, 62%; and Central or South America: 8/15, 53%). Regarding the final selection of 20 concepts, both subgroups considered all concepts to be important and relevant for inclusion in any self-reported outcome measure.

**Table 3:** Region and specialty after self-identifying with a latent class subgroup in round III of the Delphi panel.

Experts’ region and specialty		Total (n=100), n (%)^a^		Latent class subgroup 1, n (%)^b^		Latent class subgroup 2, n (%)^b^
**Region**		100 (100)		58 (58)		42 (42)
	North America	17 (17)	8 (47)		9 (53)	
	Western Europe	13 (13)	9 (69)		4 (31)	
	Southern Europe	13 (13)	8 (62)		5 (38)	
	Africa	5 (5)	4 (80)		1 (20)	
	Central/South America	15 (15)	8 (53)		7 (47)	
	Central Europe	5 (5)	4 (80)		1 (20)	
	Asia	17 (17)	5 (29)		12 (71)	
	Eastern Europe	9 (9)	7 (78)		2 (22)	
	Middle East	6 (6)	5 (83)		1 (17)	
**Specialty**		100 (100)		58 (58)		42 (42)
	General practice or internal medicine	27 (27)	17 (63)		10 (37)	
	Dentist or oral hygienist	15 (15)	11 (73)		4 (27)	
	Oncologist	19 (19)	14 (74)		5 (26)	
	Cardiovascular specialist	11 (11)	5 (45)		6 (55)	
	Respiratory	8 (8)	3 (38)		5 (63)	
	Researcher	9 (9)	4 (44)		5 (56)	
	Counselor or psychologist	6 (6)	4 (67)		2 (33)	
	Policy advisor	4 (4)	0 (0)		4 (100)	
	Charity or advocacy	1 (1)	0 (0)		1 (100)	

^a^Data for 100 experts were included, with one respondent (from South Africa) reporting not understanding the questions asked.

^b^Percentage use the n value in the “Total” column as the denominator.

### Additional Semiqualitative Assessment—Likelihood to Perceive Change

**Figure 7:**
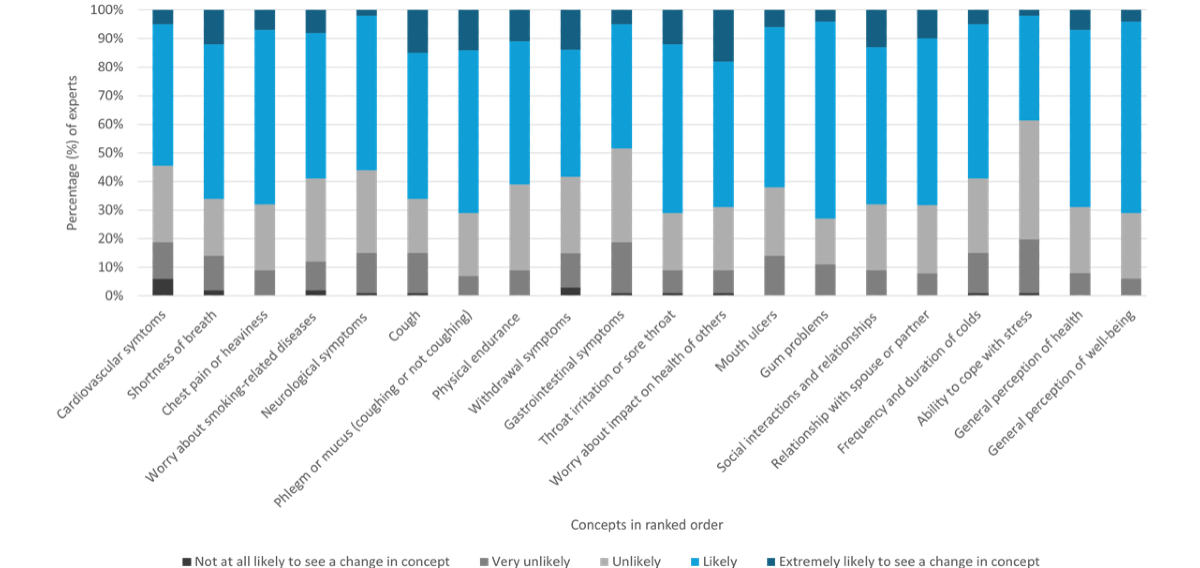
Experts’ opinions (percentage of experts) regarding likelihood of observing change in the severity of the final 20 items in round III of the Delphi panel.

In round III (in response to the question: “Now that you have seen the list of concepts collated from Rounds I & II, how likely do you think it is to see a change in this concept when a TNP user switches from a combustible TNP to an sf-TNP?”), most experts reported they would expect to find measurable levels of change in 13 out of 20 individual concepts. Furthermore, over half of the respondents stated it was “likely” or “extremely likely” that a measurable change would occur in these concepts when a TNP user switched from a combustible TNP to a sf-TNP (Figure 7). For example, majority of experts felt that it was “likely” or “extremely likely” to observe a change in gum problems (74/101, 73%), phlegm or mucus while coughing or not coughing (72/101, 71%), general perception of well-being (72/101, 71%), and throat irritation/sore throat (72/101, 71%). In addition, between 5% and 20% considered that switching was “unlikely” or “very unlikely” to result in a change in the severity of any single concept, including cardiovascular symptoms, withdrawal symptoms associated with quitting, shortness of breath, and worry about smoking-related diseases. Reasons given by experts for considering a change in a concept to be unlikely are listed in Table 4 and were mostly related to lack of perceived substantial differences between combustible TNPs and sf-TNPs.

**Table 4:** Reasons given by experts for considering a change in a concept to be unlikely for the final 20 items in round III of the Delphi panel.

Concept (number of experts responding it was unlikely to see a change)	Rationale reported by experts
Cardiovascular symptoms (n=6)	Unlikely to see substantial modifications between the combustible TNPs^a^ and sf-TNPs^b^ as tobacco or nicotine can cause cardiovascular disease Active ingredients are the same and present in both combustible TNPs and sf-TNPs products
Shortness of breath or dyspnea (n=2)	No change or no substantial modifications in switching to sf-TNPs
Worry about smoking-related diseases and impact on health in general (eg, cancer and stroke) (n=2)	Symptoms will occur with using both combustible TNPs and sf-TNPs
Worry about impact on health of others (eg, children, partner, family, and friends) (n=1)	Other dangerous chemicals in sf-TNPs
Frequency and duration of colds (n=1)	Colds are unlikely to change as they are a nuisance and not a major health threat

^a^TNPs: tobacco and/or nicotine products.

^b^sf-TNPs: smoke-free tobacco and/or nicotine products.

## Discussion

### Principal Findings

This adapted Delphi panel study was conducted to identify the health and functioning concepts that experts consider to be most relevant and useful for incorporation into a new TNP-specific health and functioning measurement instrument to assess the self-reported impact of switching from combustible TNPs to sf-TNPs [21]. We recruited a geographically and professionally representative panel of experts who routinely treated those who smoke or communicated on smoking and TNP use. They were asked to reduce and refine a preliminary list of health and functioning concepts and to rank the final list in order of importance. After 3 rounds of investigation, the initial list of 69 concepts was refined to the most important 20 they considered could be included in a new self-reported measure (Table 5). The range of final concepts reflected health and psychosocial issues associated with smoking, including respiratory and cardiovascular health, withdrawal symptoms, and worries about the impact on health of self and others.

**Table 5:** Concepts recommended and agreed after each round of the Delphi panel.

Rank	Round I (n=69), ranked in order of importance (highest to lowest)	Round II (n=36), ranked in order of importance (highest to lowest)	Round III (n=20), ranked in order of importance (highest to lowest)
1	Worry about risk of cancer	Cardiovascular symptoms (eg, palpitations, blood pressure)	Cardiovascular symptoms (eg, palpitations, blood pressure)
2	Shortness of breath or dyspnea	Shortness of breath or dyspnea	Shortness of breath or dyspnea
3	Worry about health	Worry about smoking-related diseases and impact on health in general (eg, cancer, stroke)	Chest pain or heaviness
4	Anxiety symptoms due to withdrawal	Chest pain or heaviness	Worry about smoking-related diseases and impact on health in general (eg, cancer, stroke)
5	Irritability due to withdrawal	Neurological symptoms (eg, dizziness, nausea, headaches)	Neurological symptoms (eg, dizziness, nausea, headaches)
6	Strong craving for TNP^a^ due to withdrawal	Withdrawal symptoms associated with quitting TNPs	Cough
7	Depression symptoms due to withdrawal	Cough	Phlegm or mucus while coughing or not coughing
8	Stress or tension due to withdrawal	General perception of health	Physical endurance during everyday activities and exercise
9	Ability to control moods and emotions	Phlegm or mucus while coughing or not coughing	Withdrawal symptoms associated with quitting TNPs
10	Aggression due to withdrawal	Gastrointestinal symptoms (eg, heartburn, constipation, diarrhea)	Gastrointestinal symptoms (eg, heartburn, constipation, diarrhea)
11	Worry about impact on pregnancy or fertility	Physical endurance during everyday activities and exercise	Throat irritation or sore throat
12	Concerns about the impact on the whole family	Worry about impact on health of others (eg, children, partner, family, friends)	Worry about impact on health of others (eg, children, partner, family, friends)
13	Concerns about impact on own children	Ability to cope with stress	Mouth ulcers
14	Pain or burning sensation in the teeth, gums, mouth, lips, throat, or tongue	Mouth ulcers	Gum problems (bleeding, pain, redness, swelling)
15	Bringing up phlegm or mucus while coughing or not coughing	Gum problems (bleeding, pain, redness, swelling)	Social interactions and relationships with children, family, friends, colleagues
16	Cough	Social interactions and relationships with children, family, friends, colleagues	Relationship with spouse or partner
17	Exercise capacity	Sleeping problems	Frequency and duration of colds
18	Anger due to withdrawal	General perception of well-being	Ability to cope with stress
19	Craving relief with TNP use	Ability to have or perform sexual activities	General perception of health
20	Impact on intimate relationships	Relationship with spouse or partner	General perception of well-being
21	Lack of concentration due to withdrawal	Throat irritation or sore throat	—
22	Ability to cope with stress	Frequency and duration of colds	—
23	Swollen or bleeding cheeks, lips, gums, or tongue	Ability to control moods and emotions	—
24	Ability to enjoy life	Hoarseness or change in voice	—
25	Concerns about the impact on own spouse or partner	Ability to concentrate or focus	—
26	Poor oral health in general	Weight control	—
27	Impact on relationships with children	Fatigue	—
28	Hoarseness or change in voice	Bad breath or halitosis	—
29	Ability to concentrate or focus	Feelings of self-esteem and self-respect	—
30	Yellow, gray, or black teeth	Feeling energized	—
31	Loss of tooth or teeth	Sense of smell and taste	—
32	Ability to have and maintain an erection	Yellow, gray, or black teeth	—
33	Ability to pay attention, attention span	Plaque or tartar build up	—
34	Feelings of self-esteem and self-respect	Wrinkling, yellow, and dry skin	—
35	Symptoms of fatigue	Odor on clothes, hair, or body, due to TNP use	—
36	Impact on social life	Yellow brittle nails and dry hair	—
37	Sore throat	—	—
38	Heartburn (burning sensation in the stomach)	—	—
39	Sleeping problems	—	—
40	Bad breath or halitosis	—	—
41	Improved weight control with TNP use	—	—
42	Working memory	—	—
43	Perceives a stigma due to TNP use	—	—
44	Opportunity to relax or take a break while using TNP	—	—
45	Ability to have sexual activity—with self or others	—	—
46	Acid reflux	—	—
47	Enjoyment or pleasure while using TNP	—	—
48	Plaque or tartar build up	—	—
49	Productivity at work or home	—	—
50	Throat clearing	—	—
51	Increased concentration or focus with TNP use	—	—
52	Lost sense of taste	—	—
53	Wearing away of a tooth or teeth	—	—
54	Lost sense of smell	—	—
55	Bodily pain	—	—
56	Isolated by others	—	—
57	Impact on co-workers	—	—
58	Self-isolation	—	—
59	Skin tone quality (yellow or gray fingers or face)	—	—
60	Bad odor on clothes, hair, or body due to TNP use	—	—
61	Ability to reach orgasm	—	—
62	Parenting skills	—	—
63	Enjoyment of TNP taste	—	—
64	Participation in recreational activities	—	—
65	Fitting in with others who use TNP	—	—
66	Impact on friendships	—	—
67	Concerns about safety relating to risk of fire	—	—
68	Wrinkling skin	—	—
69	Likelihood of getting a cold	—	—

^a^TNP: tobacco and/or nicotine products.

Overall, the final 20 concepts were considered both clinically relevant and important to the experts in their evaluation of the possible impact of switching to sf-TNPs or stopping smoking cigarettes. We observed that the concepts consistently ranked highly among experts focused on objectively measurable health consequences such as respiratory and cardiovascular symptoms, as well as overall physical functioning. In contrast, concepts such as the ability to cope with stress, risk of getting colds, oral health, physical appearance, social functioning, and sensory impacts (taste, sense of smell, and odor) were ranked as less important. Those concepts ranking highest reflect current evidence regarding smoking-related health outcomes and potential improvements attainable by ceasing cigarette consumption or switching to sf-TNPs [6,41-46]. The experts’ knowledge of evidence for the effect of cessation or switching on pre-existing respiratory conditions [6,43,44,47,48] may also be reflected in the ranking.

It is essential to note that the relative ranking of these concepts is likely to be different for TNP consumers compared with health care professionals. Individuals trying to stop smoking or switch to sf-TNPs recognize the respiratory and other physical health and functional benefits and understand the potential for improved quality of life by reducing cigarette consumption or stopping smoking [49,50], as well as reduced exposure of others to smoking-related harm [6,51]. They also tend to report benefits and preferences for general hygiene and smell, better oral health, and sensory improvements [6,41,43]. However, no concepts related to general physical appearance or hygiene were in the final top-ranking concepts of the current study. These results explicitly reflect the participants’ own experiences and opinions, which may differ from those of TNP users and be dependent on TNP user characteristics.

Although objective health outcomes such as cardiovascular and respiratory function were rated highly by experts throughout the study, some individuals rated psychosocial outcomes and effects on families and others (through secondhand smoke and so on) as equally important as objective physical measures. Latent class analysis in the final round of the study suggested that geographical location, cultural milieu, and professional specialism may each play a major role in these observations. Specifically, we identified a subgroup that focused mostly on physical, objectively measurable concepts (eg, cardiovascular symptoms, shortness of breath, chest pain, neurological symptoms, and cough), compared with the other subgroup, which considered both objective and subjective or emotional concepts (eg, worry about smoking-related diseases and impact on health both of self and others) to be of equal importance. These results may correspond with established patterns of perceptions and considerations among Western cultures, which may be broadly defined as individualistic (ie, driven by personal goals), whereas those from Asia and South America can be defined as collectivist (reflecting the primacy of mutual obligations among members of society) [51-53]. Furthermore, qualitative investigation of alignment with the subgroups revealed that for certain job roles (eg, counselors and policy advisors), the consumer-focused concepts were more important than for other health care professionals (eg, specialist clinicians), who were primarily concerned with objective, physical concepts. These findings have important implications for the development of a self-report measure that can be widely used and is adaptable to individual or local requirements [54]. When disseminating results based on the newly developed self-reported measure, it may be essential to tailor communications on sf-TNPs for specific audiences with different considerations of what is clinically meaningful to the experts themselves, as well as to the individual TNP user [26,33,54]. To validate the latent class findings in round III, we asked respondents to self-identify with the most relevant of the 2 subgroups. Overall, participants were able to do so readily, indicating that the 2 classes were culturally and professionally relevant, and that the new self-report measure will maximize content validity by including the range of concepts identified and ranked in round III to accommodate cultural and professional requirements and differences.

There are 3 main strengths of this study. First, we used the MaxDiff ranking to address the challenge of ranking an initial high number of concepts. Second, the incorporation of an anchor was essential in objectively identifying concepts for elimination. Third, a large panel of experts representing a broad range of professional expertise and geographies was recruited for this study. This allowed us to confirm validity of the concepts identified in the preparatory phase of the development of the new measure. It also enabled us to parse out cultural and professional subtleties that are important to consider when disseminating outcomes from the new measure. Development of an outcome measure involving both the target population and clinicians is a key component of creating a high-quality instrument [33,54]. Building this manner of collaboration and bridging between TNP users and relevant experts in this Delphi panel into the development of this new instrument would serve to enhance the potential quality and validity of the final measure by feeding into subsequent qualitative and psychometric evaluations [26].

There are also some limitations to our study. First, we did not use a strict Delphi panel process, and round I may be seen as a preliminary evaluation rather than a pure ranking and scoring exercise. Delphi panel methodology typically follows a highly structured process. In this particular case, it would have been ideal to perform additional preliminary qualitative insight work to review all the elements of a study, formally review and refine concept terminology, and test with external audiences. And, as previously mentioned, this Delphi panel focused on the experience and perceptions of experts and professionals who interact with TNP users. Consequently, the findings reported here do not have the vital context of TNP consumers opinions and perceptions. Unfortunately, it would have been impractical to address both angles in a single study with an initial list of 69 concepts. Instead, further research should consider evaluating TNP users’ perceptions and rankings in a separate study using similar methodology. In addition, there is a potential for selection bias in the study due to the exclusion of experts who specified they would not consider participating in studies by tobacco companies. This exclusion could result in a sample that is not fully representative of the broader expert community, potentially impacting the generalizability of the findings. To mitigate this, we used a diverse recruitment strategy to include experts from various regions and specialties. However, the views of nonparticipating experts might differ from those who participated, and this limitation should be considered when interpreting the results. Finally, the survey was conducted in various languages, and despite the measures taken to ensure the consistency and validity of the translated surveys, linguistic and cultural differences may have led to variations in interpretation of the survey. These variations could potentially affect the study’s findings and should be considered when interpreting the results.

### Conclusion

In conclusion, this 3-round, adapted Delphi panel identified a ranked list of 20 concepts to be considered when assessing the health and functioning status of individuals who stop smoking cigarettes or switch to using sf-TNPs or NRTs. It is to be expected that the scale of importance of each concept will vary based on the health status and concerns of an individual, whereas the ranking presented here represents a global, generalized view provided by the participating experts. In addition, the sensitivity of the concepts to accurately reflect changes in TNP use behavior will need to be determined. This would support the evaluation of the self-reported experience and impact of switching from conventional cigarettes to sf-TNPs on health risks and contribute to the regulatory and scientific evidence base for understanding both the individual and public health impacts of sf-TNPs.

## Appendix

Multimedia Appendix 1 Amended/modified Delphi panel approach.

## Abbreviations

sf-TNP: smoke-free tobacco and/or nicotine product
TNP: tobacco and/or nicotine product
NRT: nicotine replacement therapy
FDA: Food and Drug Administration

## Data Availability

The datasets generated during and/or analyzed during this study are available from the corresponding author on reasonable request.
