# Supplementary material for: International Expert Consensus on Relevant Health and Functioning Concepts to Assess in Users of Tobacco and Nicotine Products: Delphi Study
Source: JMIR Form Res. 2025 Jan 2;9:e58614. doi: 10.2196/58614 (PMC11739724; doi:10.2196/58614)
Supplement: Multimedia Appendix 1 [file formative_v9i1e58614_app1.docx]

## **Supplementary Information**

### S1. Amended/Modified Delphi Panel approach.

***Amended Round I: review and refinement of concepts***

Table S1 summarizes the rationale for refining the concepts for Round II based on the outcomes of the ABOUT-Health and Functioning development expert panel review [1]. The panel consisted of five key opinion leaders (KOLs) and five technical consultants.

The participating KOLs were subject matter experts in the fields of nicotine and smoking cessation (n=1), patient-reported outcome (PRO) evaluation and scale development (n=3), and health economics (n=1). The consultants were experts on nicotine dependence (n=1), psychometric validation (n=2), market research (n=1), and PRO development and validation (n=1). In addition, expert stakeholders from the sponsor (n=10) participated, consisting of the project team, as well as attendees representing clinical research, and scientific and medical affairs functions.

The Round I outcomes were also reviewed in the context of discussions and findings from then-ongoing concept elicitation interviews with TNP users [2, 3].

The overall conclusion from these exercises was to keep most identified concepts unchanged but with minor reformatting in some cases. For example, combining concepts relating to withdrawal into a single concept rather than eight withdrawal symptom concepts; rewording some concepts for clarity; adding concepts considered to be missing from the original list, e.g., cardiovascular risk and neurological concepts.

Table S1.

*Rationale for refinement of concepts from Round I to Round II of the Delphi Panel.*

| Round I Concept Groups and Concept  (N=69) | Final Round II Concept Groups and Concepts (N=36) and rationale for refinement |
| --- | --- |
| Concepts in **Bold** are the recommended health and functioning concepts as ranked and scored in Round I, Stage 1 (MaxDiff results matched to the outcomes of the latent group analysis). | |
| Withdrawal symptoms: Symptoms that may be experienced by TNP users when they stop using TNPs altogether or switch to a different TNP   1. **Depression symptoms due to withdrawal** 2. **Anxiety symptoms due to withdrawal** 3. **Anger due to withdrawal** 4. **Aggression due to withdrawal** 5. **Stress/tension due to withdrawal** 6. **Lack of concentration due to withdrawal** 7. **Strong craving for TNP due to withdrawal** 8. **Irritability due to withdrawal** | Withdrawal symptoms: symptoms that may be experienced by TNP users when they stop using TNPs altogether or switch to a different TNP (e.g., irritability, anxiety, depression, strong craving).   - 1. Withdrawal symptoms associated with quitting TNPs   “Withdrawal symptoms” *and* “Craving relief with TNP use” *concepts merged and reworded for clarity and conciseness based on expert panel feedback, as they considered withdrawal to be one main concept that is already measured by existing instruments and therefore a new instrument does not need details of each withdrawal symptom* |
| Poor oral health: Dental or mouth problems associated with TNP use   1. Poor oral health in general 2. **Swollen or bleeding cheeks, lips, gums, or tongue** 3. **Yellow, grey, or black teeth** 4. Plaque or tartar build up 5. Wearing away of a tooth or teeth 6. Pain or burning sensation in the teeth, gums, mouth, lips, throat, or tongue 7. Bad breath or halitosis 8. **Loss of tooth or teeth** | Oral Symptoms: how TNP use affects symptoms of oral conditions   1. Mouth ulcers (newly added concepts based on qualitative research findings and feedback from stakeholders) 2. Gum problems (bleeding, pain, redness, swelling) (“Pain or burning sensation in the teeth, gums, mouth, lips, throat, or tongue” *reworded for clarity and conciseness based on feedback from stakeholders and qualitative research findings to reflect more accurately how this oral symptom is experienced and described)* 3. Yellow, grey, or black teeth *(no change from Round I)* 4. Plaque or tartar build up (no change from Round I; based on feedback from stakeholders that it reflects “Poor oral health in general”) 5. Bad breath or halitosis (no change from Round I; based on feedback from internal stakeholders that it reflects “Poor oral health in general”)   *Feedback from stakeholders that* “Poor oral health in general” *was vague and suggest keeping specific symptoms. In addition,* “Loss of tooth or teeth” *and “*Wearing away of tooth or teeth” *were also considered vague as these symptoms that builds up over several years and have an impact on all the other areas of oral health symptoms listed and may also not necessarily be a specifically resulting from TNP use. These concepts were also not expressed by consumers from the qualitative research findings.* |
| Utility of use core set: Perceived satisfaction/benefit of TNP use   1. Enjoyment/pleasure while using TNP 2. **Craving relief with TNP use** 3. Opportunity to relax or take a break while using TNP 4. Enjoyment of TNP taste 5. Increased concentration/focus with TNP use 6. Improved weight control with TNP use 7. Fitting in with others who use TNP | “Improved weight control with TNP use” *reworded to* “Weight control” *for clarity, retained based on qualitative research findings, and moved to* “General Physical Appearance/Hygiene” group.  *A lot of the other concepts were not ranked highly as important in Round I and not to be included as core outcomes as part of the health and functioning instrument based on expert panel feedback.* “Craving relief with TNP use” *was further merged with* “Withdrawal symptoms associated with quitting TNPs” *based on expert panel feedback as it is considered a part of myriad of withdrawal symptoms* |
| Sleep and fatigue symptoms associated with TNP use   1. **Symptoms of fatigue** 2. Sleeping problems | *Both concepts retained based on qualitative research findings and moved to* “Other Physical Health Symptoms” group. |
| Respiratory symptoms: Symptoms associated with TNP-related respiratory infections or disease   1. **Bringing up phlegm or mucus while coughing or not coughing** 2. **Shortness of breath or dyspnea** 3. **Cough** 4. Throat clearing 5. Likelihood of getting a cold | Respiratory Symptoms – how TNP use affects symptoms of respiratory infections or conditions   1. Phlegm or mucus while coughing or not coughing *(no change from Round I)* 2. Shortness of breath or dyspnea *(no change from Round I)* 3. Cough *(no change from Round I)* 4. Chest pain/heaviness *(newly added concept based on qualitative research findings)* 5. Frequency and duration colds *(retained and reworded for clarity to include frequency and duration of cold based on qualitative research findings)* |
| General health perceptions core set: TNP users’ concerns about how TNP use impacts their health   1. Worry about health 2. **Worry about risk of cancer** 3. **Worry about impact on pregnancy or fertility** 4. Concerns about safety relating to risk of fire | General Health Perceptions – how TNP use affects TNP users’ perceptions of their health status   1. General perception of health (newly added concept based on expert panel feedback and qualitative research findings) 2. General perception of well-being (newly added concept based on expert panel feedback and qualitative research findings)   Worries about health risks – TNP users’ concerns about the impact of TNP use on their health and health of others   1. Worry about smoking-related diseases and impact on health in general (e.g., cancer, stroke, lung disease, heart disease) (merged and reworded based on stakeholders’ feedback and qualitative research findings to capture examples of major health risks expressed by consumers) 2. Worry about impact on health of others (e.g., children, partner, family, friends)   “Concerns about impact on own children,” “Concerns about the impact on own spouse or partner,” *and* “Concerns about the impact on the whole family” from “Social functioning core set” *merged and reworded* *based on internal stakeholders’ feedback and qualitative research findings to capture examples of major concerns expressed by consumers on impact on others.* |
| Emotional core set: Emotional states impacted by the use, switching, or cessation of TNPs   1. Ability to control moods and emotions 2. **Ability to enjoy life** 3. **Feelings of self-esteem and self-respect** 4. **Ability to cope with stress** | Emotional Functioning – how TNP use affects TNP users’ emotional states   1. Ability to control moods and emotions *(no change from Round I)* 2. Feelings of self-esteem and self-respect *(no change from Round I)* 3. Ability to cope with stress *(no change from Round I)*   *Based on internal stakeholders’ and expert panel feedback,* “Ability to enjoy life” *was felt to be better captured by* “General perception of well-being,” *which covers aspects of life satisfaction, etc.* |
| Other symptoms related to TNP use   1. Hoarseness or change in voice 2. Sore throat 3. Lost sense of taste 4. Lost sense of smell 5. Bodily pain 6. Acid reflux 7. Heartburn (burning sensation in the stomach) | Other Physical Health Symptoms – how TNP use affects other symptoms of physical health conditions   1. Cardiovascular symptoms (e.g., palpitations, blood pressure) *(newly added concept based on qualitative research findings)* 2. Neurological symptoms (e.g., dizziness, nausea, headaches) (newly added concept based on qualitative research findings) 3. Gastrointestinal symptoms (e.g., heartburn, constipation, diarrhea) *(newly added concept based on qualitative research findings)* 4. Fatigue *(no change from Round I; reworded for clarity and moved to this concept group)* 5. Sleeping problems (no change from Round I; retained based on qualitative research findings) 6. Throat irritation/sore throat *(*“Sore throat” *reworded for clarity based on qualitative research findings)* 7. Hoarseness or change in voice *(no change from Round I)* 8. Sense of smell and taste (“Lost sense of taste” *and* “Lost sense of smell” *reworded and* *merged for clarity and based on qualitative research findings)* |
| Functioning core set: Instrumental, physical, cognitive and recreational activities of daily living that may be impacted by the use, switching, or cessation of TNPs   1. Productivity at work or home 2. Parenting skills 3. **Exercise capacity** 4. Working memory 5. **Ability to concentrate or focus** 6. **Ability to pay attention, attention span** 7. **Ability to have and maintain an erection** 8. Ability to have sexual activity – with self or others 9. Ability to reach orgasm 10. Participation in recreational activities | Functioning – how TNP use affects instrumental, physical, cognitive, sexual, and recreational activities of daily living   1. Physical endurance during everyday activities and exercise *(*“Exercise capacity” *reworded for clarity and based on based on qualitative research findings)* 2. Feeling energized (newly added concept based on qualitative research findings) 3. Ability to concentrate or focus *(no change from Round I;* “Ability to pay attention, attention span” *also considered to be part of this concept based on stakeholders’ feedback and qualitative research findings*) 4. Ability to have/perform sexual activities *(*“Ability to have and maintain an erection,” “Ability to have sexual activity – with self or others,” *and* “Ability to reach orgasm” *reworded and merged for clarity and based on based on qualitative research findings)* |
| Social functioning core set: How use, switching, or cessation of TNPs affect a TNP user’s social activities and relationships   1. Perceives a stigma due to TNP use 2. Self-isolation 3. Isolated by others 4. Impact on social life 5. **Impact on relationships with children** 6. **Impact on intimate relationships** 7. Impact on friendships 8. **Concerns about impact on own children** 9. Impact on co-workers 10. **Concerns about the impact on own spouse or partner** 11. **Concerns about the impact on the whole family** | Social Functioning – how TNP use affects a TNP user’s social roles, activities and relationships   1. Social interactions and relationships with children, family, friends, colleagues (newly added concept to capture main aspects of social functioning concepts, including “impact on relationships with children” based on stakeholders’ feedback and qualitative research findings) 2. Relationship with spouse/partner *(*“Impact on intimate relationships” *reworded for clarity based on qualitative research findings)*   “Concerns about impact on own children,” “Concerns about the impact on own spouse or partner,” *and* “Concerns about the impact on the whole family” *merged and reworded to* “Worry about impact on health of others (e.g., children, partner, family, friends)” *and moved to* “Worries about health risks” *group based on stakeholders’ feedback and qualitative research findings to capture examples of major concerns expressed by consumers on impact on others.* |
| Other symptoms related to general hygiene or appearance   1. Bad odor on clothes, hair, or body, due to TNP use 2. Skin tone quality (yellow/grey fingers or face) 3. Wrinkling skin | General Physical Appearance/Hygiene – how TNP use affects general symptoms related to appearance or hygiene   1. Weight control *(*“Improved weight control with TNP use” *reworded to* “Weight control” *for clarity, retained based on qualitative research findings and moved from* “Utility of use core set”*)* 2. Odor on clothes, hair, or body, due to TNP use (no change from Round I; reworded for clarity based on stakeholders’ feedback). 3. Wrinkling, yellow and dry skin *(*“Skin tone quality (yellow/grey fingers or face)” and “Wrinkling skin” *merged and reworded for clarity based on qualitative research findings)* 4. Yellow brittle nails and dry hair *(reworded/newly added concepts)* *(*“Skin tone quality (yellow/grey fingers or face)” *expanded and reworded for clarity based on qualitative research findings).* |

#### References

1. Afolalu EF, Spies E, Bacso A, Clerc E, Abetz-Webb L, Gallot S, et al. Impact of tobacco and/or nicotine products on health and functioning: a scoping review and findings from the preparatory phase of the development of a new self-report measure. Harm Reduct J. Jul 30, 2021;18(1):79. [doi: 10.1186/s12954-021-00526-z] [PMID: 34330294]

2. Afolalu EF, Bacso A, Abetz-Webb L, Clerc E, Spies E, Chrea C. The process of developing a new outcome measure to assess the impact of tobacco and/or nicotine-containing products on health and functioning. 26th Annual Meeting Society for Research on Nicotine and Tobacco (SRNT); New Orleans, USA2020.

3. Afolalu EF, Lanar S, Abetz-Webb L, Bacso A, Blankenspoor M, Koochaki P, et al. Qualitative health and functioning concept elicitation interviews in Japanese users of heated tobacco products. 27th Annual Meeting Society for Research on Nicotine and Tobacco (SRNT); Online2020.
